# (*E*)-2-Meth­oxy-*N*′-(4-methoxy­benzyl­idene)benzohydrazide

**DOI:** 10.1107/S1600536809039713

**Published:** 2009-10-03

**Authors:** Guo-Biao Cao

**Affiliations:** a Department of Chemistry, Ankang University, Ankang Shanxi 725000, People’s Republic of China

## Abstract

The mol­ecule of the title compound, C_16_H_16_N_2_O_3_, displays an *E* configuration about the C=N bond. The dihedral angle between the two benzene rings is 99.0 (2)°. In the crystal structure, mol­ecules are linked through inter­molecular N—H⋯O hydrogen bonds, forming chains running along the *b* axis.

## Related literature

For examples of the crystal structures of hydrazone compounds, see: Mohd Lair *et al.* (2009 ▶); Fun *et al.* (2008 ▶); Li & Ban (2009 ▶); Zhu *et al.* (2009 ▶); Yang (2007 ▶); You *et al.* (2008 ▶). For the hydrazone compounds we have reported previously, see: Qu *et al.* (2008 ▶); Yang *et al.* (2008 ▶); Cao & Lu (2009*a* ▶,*b* ▶); Qu & Cao (2009 ▶); Cao & Wang (2009 ▶).
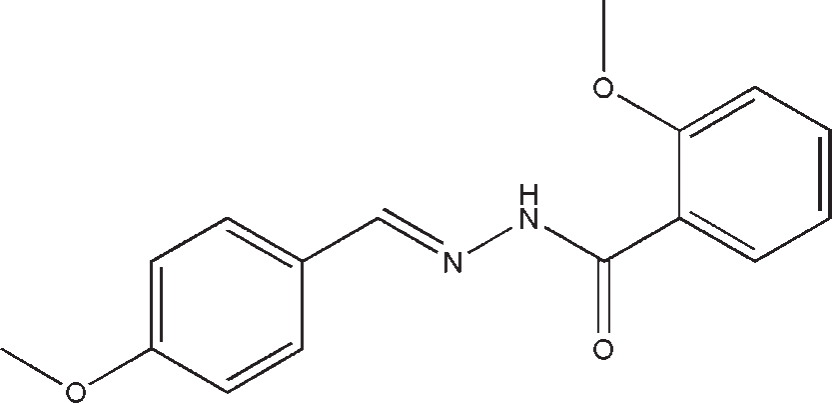


## Experimental

### 

#### Crystal data

C_16_H_16_N_2_O_3_
*M* *_r_* = 284.31
Orthorhombic, 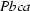

*a* = 14.990 (1) Å
*b* = 8.076 (1) Å
*c* = 24.122 (2) Å
*V* = 2920.2 (5) Å^3^
*Z* = 8
Mo *K*α radiation
μ = 0.09 mm^−1^
*T* = 298 K
0.17 × 0.15 × 0.15 mm

#### Data collection

Bruker SMART CCD area-detector diffractometer
Absorption correction: multi-scan (*SADABS*; Bruker, 2001 ▶) *T*_min_ = 0.985, *T*_max_ = 0.987
16039 measured reflections
3011 independent reflections
1225 reflections with *I* > 2σ(*I*)
*R*_int_ = 0.117

#### Refinement

*R*[*F* ^2^ > 2σ(*F* ^2^)] = 0.050
*wR*(*F* ^2^) = 0.149
*S* = 0.92
3011 reflections
196 parameters
1 restraint
H atoms treated by a mixture of independent and constrained refinement
Δρ_max_ = 0.17 e Å^−3^
Δρ_min_ = −0.15 e Å^−3^

### 

Data collection: *SMART* (Bruker, 2007 ▶); cell refinement: *SAINT* (Bruker, 2007 ▶); data reduction: *SAINT*; program(s) used to solve structure: *SHELXTL* (Sheldrick, 2008 ▶); program(s) used to refine structure: *SHELXTL*; molecular graphics: *SHELXTL*; software used to prepare material for publication: *SHELXTL*.

## Supplementary Material

Crystal structure: contains datablocks global, I. DOI: 10.1107/S1600536809039713/rz2367sup1.cif

Structure factors: contains datablocks I. DOI: 10.1107/S1600536809039713/rz2367Isup2.hkl

Additional supplementary materials:  crystallographic information; 3D view; checkCIF report

## Figures and Tables

**Table 1:** Hydrogen-bond geometry (Å, °)

*D*—H⋯*A*	*D*—H	H⋯*A*	*D*⋯*A*	*D*—H⋯*A*
N2—H2*A*⋯O2^i^	0.899 (10)	2.093 (15)	2.940 (3)	157 (3)

Symmetry code: (i) 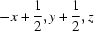.

**Fig. 1.**
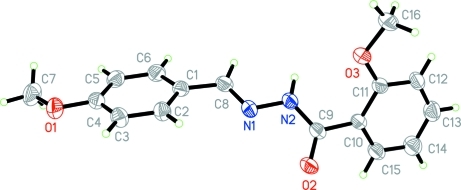
The molecular structure of the title compound with ellipsoids drawn at the 30% probability level.

**Fig. 2.**
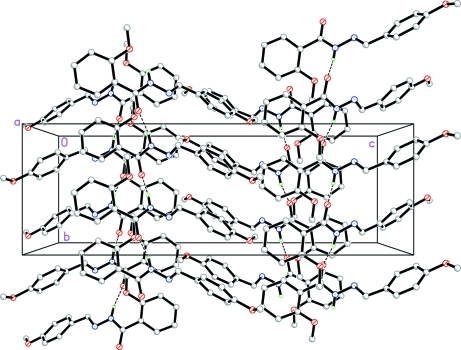
The molecular packing of the title compound, viewed along the a axis. Hydrogen bonds are drawn as dashed lines.

## supplementary crystallographic information

### Comment

Study on the crystal structures of hydrazone derivatives is an interesting topic in structural chemistry. Recently, the crystal structures of a number of hydrazone compounds have been reported (Mohd Lair *et al.*, 2009; Fun *et al.*, 2008; Li & Ban, 2009; Zhu *et al.*, 2009; Yang, 2007; You *et al.*, 2008). As a continuation of our work in this area (Qu *et al.*, 2008; Yang *et al.*, 2008; Cao & Lu, 2009a,b; Qu & Cao, 2009; Cao & Wang, 2009), the title new hydrazone compound, derived from the reaction of 4-methoxybenzaldehyde with an equimolar quantity of 2-methoxybenzohydrazide, is reported.

The molecule of the title compound (Fig. 1) displays an *E* configuration about the C═N bond. The dihedral angle between the two benzene rings is 99.0 (2)°. In the crystal structure, molecules are linked through intermolecular N—H···O hydrogen bonds (Table 1) to form chains running along the *b* axis (Fig. 2).

### Experimental

The title compound was prepared by refluxing 4-methoxybenzaldehyde (0.1 mmol, 13.6 mg) with 2-methoxybenzohydrazide (0.1 mmol, 16.6 mg) in methanol (20 ml). Colourless block-like crystals were formed by slow evaporation of the solution in air.

### Refinement

H2A was located in a difference Fourier map and refined isotropically, with the N—H distance restrained to 0.90 (1) Å. The other H atoms were placed in idealized positions and constrained to ride on their parent atoms, with C—H distances of 0.93-0.96 Å, and with *U*_iso_(H) set at 1.2*U*_eq_(C) and 1.5*U*_eq_(methyl C).

### Crystal data

**Table:**

C_16_H_16_N_2_O_3_	*F*(000) = 1200
*M**_r_* = 284.31	*D*_x_ = 1.293 Mg m^−^^3^
Orthorhombic, *P**b**c**a*	Mo *K*α radiation, λ = 0.71073 Å
Hall symbol: -P 2ac 2ab	Cell parameters from 687 reflections
*a* = 14.990 (1) Å	θ = 2.6–24.5°
*b* = 8.076 (1) Å	µ = 0.09 mm^−^^1^
*c* = 24.122 (2) Å	*T* = 298 K
*V* = 2920.2 (5) Å^3^	Block, colourless
*Z* = 8	0.17 × 0.15 × 0.15 mm

### Data collection

**Table:**

Bruker SMART CCD area-detector diffractometer	3011 independent reflections
Radiation source: fine-focus sealed tube	1225 reflections with *I* > 2σ(*I*)
graphite	*R*_int_ = 0.117
ω scans	θ_max_ = 26.5°, θ_min_ = 1.7°
Absorption correction: multi-scan (*SADABS*; Bruker, 2001)	*h* = −18→14
*T*_min_ = 0.985, *T*_max_ = 0.987	*k* = −10→9
16039 measured reflections	*l* = −29→30

### Refinement

**Table:**

Refinement on *F*^2^	Secondary atom site location: difference Fourier map
Least-squares matrix: full	Hydrogen site location: inferred from neighbouring sites
*R*[*F*^2^ > 2σ(*F*^2^)] = 0.050	H atoms treated by a mixture of independent and constrained refinement
*wR*(*F*^2^) = 0.149	*w* = 1/[σ^2^(*F*_o_^2^) + (0.0609*P*)^2^] where *P* = (*F*_o_^2^ + 2*F*_c_^2^)/3
*S* = 0.92	(Δ/σ)_max_ = 0.001
3011 reflections	Δρ_max_ = 0.17 e Å^−^^3^
196 parameters	Δρ_min_ = −0.15 e Å^−^^3^
1 restraint	Extinction correction: *SHELXTL* (Sheldrick, 2008), Fc^*^=kFc[1+0.001xFc^2^λ^3^/sin(2θ)]^-1/4^
Primary atom site location: structure-invariant direct methods	Extinction coefficient: 0.0064 (9)

### Special details

**Table:**

Geometry. All esds (except the esd in the dihedral angle between two l.s. planes) are estimated using the full covariance matrix. The cell esds are taken into account individually in the estimation of esds in distances, angles and torsion angles; correlations between esds in cell parameters are only used when they are defined by crystal symmetry. An approximate (isotropic) treatment of cell esds is used for estimating esds involving l.s. planes.
Refinement. Refinement of F^2^ against ALL reflections. The weighted R-factor wR and goodness of fit S are based on F^2^, conventional R-factors R are based on F, with F set to zero for negative F^2^. The threshold expression of F^2^ > 2sigma(F^2^) is used only for calculating R-factors(gt) etc. and is not relevant to the choice of reflections for refinement. R-factors based on F^2^ are statistically about twice as large as those based on F, and R- factors based on ALL data will be even larger.

### Fractional atomic coordinates and isotropic or equivalent isotropic displacement parameters (Å^2^)

**Table:**

	*x*	*y*	*z*	*U*_iso_*/*U*_eq_	
N1	0.24763 (16)	0.2770 (3)	0.63202 (9)	0.0545 (7)	
N2	0.22280 (16)	0.3524 (3)	0.68158 (10)	0.0538 (7)	
O1	0.44582 (16)	0.0703 (3)	0.41056 (8)	0.0775 (7)	
O2	0.15343 (14)	0.1210 (3)	0.71066 (8)	0.0674 (6)	
O3	0.12187 (13)	0.6010 (3)	0.73232 (9)	0.0704 (7)	
C1	0.3439 (2)	0.2823 (3)	0.55389 (11)	0.0513 (8)	
C2	0.2946 (2)	0.1755 (4)	0.52098 (12)	0.0596 (9)	
H2	0.2364	0.1493	0.5310	0.072*	
C3	0.3305 (2)	0.1079 (4)	0.47389 (13)	0.0629 (9)	
H3	0.2962	0.0374	0.4521	0.076*	
C4	0.4177 (2)	0.1438 (4)	0.45845 (12)	0.0591 (9)	
C5	0.4675 (2)	0.2488 (4)	0.49037 (12)	0.0638 (9)	
H5	0.5260	0.2732	0.4806	0.077*	
C6	0.4299 (2)	0.3188 (4)	0.53754 (12)	0.0628 (9)	
H6	0.4637	0.3921	0.5586	0.075*	
C7	0.5366 (2)	0.0952 (5)	0.39380 (14)	0.0938 (12)	
H7A	0.5468	0.2109	0.3873	0.141*	
H7B	0.5480	0.0342	0.3604	0.141*	
H7C	0.5759	0.0571	0.4225	0.141*	
C8	0.3105 (2)	0.3490 (4)	0.60596 (12)	0.0565 (8)	
H8	0.3353	0.4453	0.6204	0.068*	
C9	0.17680 (19)	0.2648 (4)	0.71898 (12)	0.0518 (8)	
C10	0.15877 (18)	0.3466 (3)	0.77329 (11)	0.0473 (7)	
C11	0.13233 (17)	0.5107 (4)	0.77954 (13)	0.0518 (8)	
C12	0.11833 (19)	0.5735 (4)	0.83241 (14)	0.0632 (9)	
H12	0.1021	0.6838	0.8370	0.076*	
C13	0.1284 (2)	0.4732 (5)	0.87795 (14)	0.0754 (10)	
H13	0.1197	0.5170	0.9132	0.091*	
C14	0.1508 (2)	0.3111 (5)	0.87232 (14)	0.0771 (10)	
H14	0.1560	0.2432	0.9033	0.092*	
C15	0.1658 (2)	0.2486 (4)	0.81986 (13)	0.0636 (9)	
H15	0.1811	0.1376	0.8159	0.076*	
C16	0.0725 (2)	0.7525 (4)	0.73634 (14)	0.0903 (12)	
H16A	0.0178	0.7328	0.7559	0.135*	
H16B	0.0595	0.7929	0.6998	0.135*	
H16C	0.1073	0.8332	0.7560	0.135*	
H2A	0.2470 (19)	0.452 (2)	0.6882 (13)	0.080*	

### Atomic displacement parameters (Å^2^)

**Table:**

	*U*^11^	*U*^22^	*U*^33^	*U*^12^	*U*^13^	*U*^23^
N1	0.0619 (17)	0.0495 (16)	0.0521 (15)	0.0013 (13)	0.0033 (13)	−0.0108 (13)
N2	0.0596 (17)	0.0448 (16)	0.0572 (15)	−0.0048 (13)	0.0059 (13)	−0.0116 (14)
O1	0.0876 (18)	0.0791 (17)	0.0657 (14)	0.0123 (13)	0.0205 (13)	−0.0048 (12)
O2	0.0754 (15)	0.0436 (14)	0.0831 (15)	−0.0082 (11)	0.0145 (12)	−0.0180 (12)
O3	0.0831 (16)	0.0475 (13)	0.0806 (15)	0.0149 (11)	0.0209 (13)	0.0047 (12)
C1	0.058 (2)	0.0453 (18)	0.0505 (17)	0.0018 (15)	−0.0028 (15)	0.0002 (15)
C2	0.055 (2)	0.064 (2)	0.0597 (19)	−0.0010 (16)	0.0036 (16)	−0.0066 (17)
C3	0.064 (2)	0.066 (2)	0.0587 (19)	−0.0018 (17)	−0.0036 (17)	−0.0125 (17)
C4	0.071 (2)	0.052 (2)	0.0545 (19)	0.0117 (17)	0.0061 (17)	0.0034 (16)
C5	0.062 (2)	0.063 (2)	0.067 (2)	−0.0049 (17)	0.0077 (18)	0.0079 (18)
C6	0.068 (2)	0.060 (2)	0.060 (2)	−0.0112 (17)	−0.0024 (17)	−0.0026 (17)
C7	0.089 (3)	0.108 (3)	0.085 (2)	0.030 (2)	0.036 (2)	0.014 (2)
C8	0.067 (2)	0.0452 (19)	0.0571 (19)	−0.0020 (16)	−0.0020 (17)	−0.0046 (16)
C9	0.0468 (18)	0.0441 (19)	0.065 (2)	0.0042 (15)	0.0003 (15)	−0.0089 (17)
C10	0.0449 (17)	0.0409 (18)	0.0560 (18)	0.0016 (13)	0.0002 (14)	−0.0063 (15)
C11	0.0481 (19)	0.0448 (19)	0.062 (2)	−0.0015 (14)	0.0059 (15)	−0.0028 (16)
C12	0.058 (2)	0.054 (2)	0.078 (2)	0.0001 (15)	0.0160 (18)	−0.0192 (19)
C13	0.082 (3)	0.080 (3)	0.064 (2)	−0.004 (2)	0.0088 (19)	−0.018 (2)
C14	0.092 (3)	0.077 (3)	0.062 (2)	0.013 (2)	0.0017 (19)	0.002 (2)
C15	0.071 (2)	0.053 (2)	0.067 (2)	0.0123 (17)	0.0026 (17)	−0.0045 (18)
C16	0.089 (3)	0.057 (2)	0.124 (3)	0.031 (2)	0.033 (2)	0.016 (2)

### Geometric parameters (Å, °)

**Table:**

N1—C8	1.273 (3)	C6—H6	0.9300
N1—N2	1.392 (3)	C7—H7A	0.9600
N2—C9	1.338 (3)	C7—H7B	0.9600
N2—H2A	0.899 (10)	C7—H7C	0.9600
O1—C4	1.366 (3)	C8—H8	0.9300
O1—C7	1.434 (4)	C9—C10	1.492 (4)
O2—C9	1.230 (3)	C10—C15	1.378 (4)
O3—C11	1.362 (3)	C10—C11	1.391 (4)
O3—C16	1.432 (3)	C11—C12	1.388 (4)
C1—C6	1.381 (4)	C12—C13	1.373 (4)
C1—C2	1.386 (4)	C12—H12	0.9300
C1—C8	1.455 (4)	C13—C14	1.359 (5)
C2—C3	1.370 (4)	C13—H13	0.9300
C2—H2	0.9300	C14—C15	1.381 (4)
C3—C4	1.390 (4)	C14—H14	0.9300
C3—H3	0.9300	C15—H15	0.9300
C4—C5	1.367 (4)	C16—H16A	0.9600
C5—C6	1.390 (4)	C16—H16B	0.9600
C5—H5	0.9300	C16—H16C	0.9600
			
C8—N1—N2	115.0 (2)	N1—C8—C1	120.8 (3)
C9—N2—N1	119.1 (2)	N1—C8—H8	119.6
C9—N2—H2A	124 (2)	C1—C8—H8	119.6
N1—N2—H2A	116 (2)	O2—C9—N2	122.4 (3)
C4—O1—C7	118.1 (3)	O2—C9—C10	120.7 (3)
C11—O3—C16	117.4 (2)	N2—C9—C10	116.9 (3)
C6—C1—C2	117.8 (3)	C15—C10—C11	118.7 (3)
C6—C1—C8	119.3 (3)	C15—C10—C9	116.6 (3)
C2—C1—C8	122.8 (3)	C11—C10—C9	124.6 (3)
C3—C2—C1	120.9 (3)	O3—C11—C12	123.7 (3)
C3—C2—H2	119.5	O3—C11—C10	116.9 (3)
C1—C2—H2	119.5	C12—C11—C10	119.4 (3)
C2—C3—C4	120.5 (3)	C13—C12—C11	120.2 (3)
C2—C3—H3	119.7	C13—C12—H12	119.9
C4—C3—H3	119.7	C11—C12—H12	119.9
O1—C4—C5	125.3 (3)	C14—C13—C12	121.0 (3)
O1—C4—C3	115.3 (3)	C14—C13—H13	119.5
C5—C4—C3	119.5 (3)	C12—C13—H13	119.5
C4—C5—C6	119.5 (3)	C13—C14—C15	119.0 (3)
C4—C5—H5	120.2	C13—C14—H14	120.5
C6—C5—H5	120.2	C15—C14—H14	120.5
C1—C6—C5	121.7 (3)	C10—C15—C14	121.6 (3)
C1—C6—H6	119.2	C10—C15—H15	119.2
C5—C6—H6	119.2	C14—C15—H15	119.2
O1—C7—H7A	109.5	O3—C16—H16A	109.5
O1—C7—H7B	109.5	O3—C16—H16B	109.5
H7A—C7—H7B	109.5	H16A—C16—H16B	109.5
O1—C7—H7C	109.5	O3—C16—H16C	109.5
H7A—C7—H7C	109.5	H16A—C16—H16C	109.5
H7B—C7—H7C	109.5	H16B—C16—H16C	109.5

### Hydrogen-bond geometry (Å, °)

**Table:**

*D*—H···*A*	*D*—H	H···*A*	*D*···*A*	*D*—H···*A*
N2—H2A···O2^i^	0.90 (1)	2.09 (2)	2.940 (3)	157 (3)

Symmetry codes: (i) −*x*+1/2, *y*+1/2, *z*.
